# Racial Disparities in Door-to-Clinician Time for Cardiac Chest Pain in the Emergency Department

**DOI:** 10.5811/westjem.48835

**Published:** 2026-02-07

**Authors:** Emad Awad, Shilpa Raju, Hesham Alsayyed, Ramsey Issa, Jeffrey Druck

**Affiliations:** * University of Utah, School of Medicine, Department of Emergency Medicine, Salt Lake City, Utah; † Walden University, College of Nursing, Minneapolis, Minnesota; ‡ University of Utah, Department of Materials Science and Engineering, Salt Lake City, Utah

## Abstract

**Introduction:**

Timely evaluation in the emergency department (ED) is critical for patients with cardiac chest pain. Although racial disparities in ED wait times have been reported, few studies have focused specifically on cardiac-related presentations. In this study we assessed racial and ethnic disparities in ED door-to-clinician time for cardiac chest pain.

**Methods:**

We conducted a retrospective analysis of adult ED visits for cardiac chest pain (2019–2025) at a tertiary-care academic hospital. Patients ≥ 18 years of age were included. Race/ethnicity was categorized as White, Hispanic/Latino, Black, Native American, Asian, or other/unknown. Multivariable generalized linear modeling assessed the association between race/ethnicity and door-to-clinician time, adjusting for demographics and clinical variables.

**Results:**

The study included 3,925 patients. The overall median door-to-clinician time was 15.9 minutes (interquartile range 8.0–36.0). In unadjusted bivariate analyses, significant differences were observed across racial and ethnic groups (P < .001). Native American patients experienced the longest delays (23.8 minutes [13.9–49.8]), followed by Asian (18.6 minutes [8.4–36.5]) and Hispanic/Latino patients (17.1 minutes [9.3–43.7]). In contrast, White and Black patients had shorter median wait times of 14.9 minutes [7.1–33.9] and 15.0 minutes [8.8–38.7], respectively. After adjustment for age, sex, triage acuity, clinician type, and initial vital signs, Hispanic/Latino patients waited 18.2 minutes vs 14.9 minutes for White patients (absolute +3.3 minutes; 22% longer; relative risk 1.22, 95% CI, 1.09–1.36, P < .001). Adjusted times were also higher for Black (16.5 minutes), Native American (17.7 minutes), and Asian patients (15.1 minutes), but differences were not statistically significant.

**Conclusion:**

Hispanic/Latino patients with cardiac chest pain experienced a 22% longer ED wait time than White patients. Our findings highlight the need for targeted interventions and multisite research to ensure equitable, timely care for all patients with acute cardiac conditions.

## INTRODUCTION

Acute chest pain is one of the most common presenting complaints in the emergency department (ED).^1^–^3^ It may indicate serious conditions such as acute coronary syndrome (ACS) or myocardial injury. Timely evaluation in the ED is essential for the early diagnosis and management of ACS.^2^,^4^ However, not all patients presenting with chest pain receive equally timely care. Emerging evidence suggests that timeliness of evaluation and management may vary based on patient sex or race.^3^,^5^ Racial and ethnic differences in ED management have been well documented. Multiple studies report that Black and Hispanic/Latino patients are more likely to be assigned lower Emergency Severity Index (ESI) levels and experience longer door-to- clinician times compared to White patients.^6^–^12^

These delays may result in missed opportunities for early risk stratification, delayed diagnostics or treatment and, ultimately, worse clinical outcomes, thereby contributing to persistent health disparities. For example, data from the National Hospital Ambulatory Medical Care Survey found that Black patients with chest pain or dyspnea had significantly longer ED wait times than White patients. A recent analysis of over 310,000 ED visits showed that Hispanic/Latino and Black patients experienced longer median wait times than non-Hispanic White patients. Although some of these differences were attributed to factors such as mode of arrival and ESI, 12–27% of the disparities remained unexplained, potentially reflecting systemic bias or unmeasured confounders.^13^ Similarly, a retrospective analysis of 28,705 ED visits across 17 sites found that Black patients with chest pain experienced significantly longer wait times for evaluation by emergency clinicians compared to White patients.^14^ Banco et al (2022) also reported that Black patients received less timely and less aggressive evaluation for chest pain compared to their White counterparts.^3^ These patterns of unequal care may delay timely interventions, contributing to higher rates of missed or late diagnoses and worse cardiovascular outcomes.

Despite well-documented racial differences in ED wait times, gaps remain in understanding disparities among patients with confirmed cardiac chest pain. Prior studies often combined cardiac and non-cardiac presentations, risking misclassification bias. Additionally, most data come from East Coast urban safety-net hospitals, limiting generalizability. Our study addresses these gaps by focusing on confirmed cardiac chest pain in an understudied western US setting. The objective was to compare door-to-clinician times across racial and ethnic groups among adults presenting to a single, tertiary-care, urban, academic ED, to identify potential disparities in timely care delivery.

## METHODS

### Study Design and Settings

In this retrospective observational cohort study we analyzed electronic health record (EHR) data from adult patients presenting to the ED with cardiac-related chest pain between 2019–2025. The study was conducted at a tertiary-care, urban, academic ED in the western United States that serves a diverse patient population with approximately 62,000 annual visits. The ED provides comprehensive emergency care, including specialized cardiac services, and employs standardized triage protocols based on the ESI and chief complaint. For chest pain, triage pathways target a 12-lead electrocardiogram (ECG) within 10 minutes of arrival; additionally, standing nursing orders permit ECG and initial laboratory testing before clinician evaluation.

**Population Health Research Capsule**

What do we already know about this issue?

*Emergency department wait-time disparities by race/ethnicity are documented, but cardiac chest-pain–specific delays and adjusted comparisons across groups remain understudied*.

What was the research question?

*Do door-to-clinician times for cardiac chest pain differ by race/ethnicity in a tertiary ED?*

What was the major finding of the study?

*Hispanic patients with cardiac chest pain waited 3.3 minutes longer (18.2 vs. 14.9 minutes, 22%) than White patients for ED clinician evaluation (RR 1.22, 95% CI 1.09–1.36, p<0.001)*.

How does this improve population health?

*Quantifying inequities in time-to-evaluation identifies targets for ED workflow and triage reforms, guiding interventions for equitable cardiac care*.

### Study Population

We included adult patients (≥18 years) who presented to the ED with cardiac symptoms, met the criteria for cardiac chest pain defined by elevated high-sensitivity cardiac troponin T (hs-cTnT) levels above sex-specific 99th percentile upper reference limits (≥14 nanograms per liter [ng/L] for females and ≥22 ng/L for males), consistent with institutional and guideline standards. An ACS diagnosis was confirmed using a combination of emergency physician documentation, ECG findings (eg, new ST-segment changes, T-wave inversions, and OMI equivalents such as new left bundle branch block, bifascicular block, or posterior myocardial infarction [MI] patterns), and *International Classification of Diseases, 10^th^ Modification* codes (eg, I21.x for acute MI, I20.0 for unstable angina). This case definition reflects retrospective adjudication based on the ED workup, not triage impressions. Patients without ischemic ECG changes but with elevated troponin, including those with a significant delta rise on repeat testing, were also included to capture the full spectrum of myocardial injury.

We excluded cases with troponin elevation due to non-cardiac causes (eg, sepsis, renal failure, pulmonary embolism, myocarditis, pericarditis, tachyarrhythmia-related demand), old or non-ischemic ECG changes, and ESI levels other than 2 or 3. We excluded five ESI level 4 cases due to their low representation, which would not meaningfully have impacted disparity estimates. We also excluded patients who eloped, left against medical advice (AMA), or had missing key data.

### Data Source and Variables

We obtained data for this study from the Epic EHR system (Epic Corporation, Verona, WI). The data included de-identified records of patients who visited the ED. The database contains comprehensive information, including patient demographics and clinical variables. The primary outcome measure was door-to-clinician time, defined as the number of minutes from a patient’s arrival at the ED to their initial evaluation by a clinician. The primary exposure variable was race/ethnicity as documented in the EHR. Categories included the following: White; Hispanic/Latino; Black; Native American (American Indian/Alaska Native); Asian American; and other (including “refused to say” or “unknown”). Other covariates included age (measured continuously in years), sex (categorized as male or female), and triage acuity. We also included patients’ initial vital signs recorded at triage and clinician type, as well as whether the first evaluation was by an advanced care practitioner (ACP) or emergency physician.

This retrospective chart review followed key methodological standards recommended by Worster et al^15^ as follows: clearly defined inclusion and exclusion criteria; standardized and piloted data abstraction forms; use of trained and blinded abstractors; inter-rater reliability checks on a subset of charts; and routine monitoring of data quality throughout the abstraction process. This retrospective chart review was determined exempt by the University of Utah Institutional Review Board.

### Data Analysis

We used descriptive statistics to summarize patient demographic and clinical characteristics, as well as door-to-clinician times across racial and ethnic groups. Continuous variables were reported as means with standard deviations for normally distributed data or medians with interquartile ranges for non-normally distributed data. Categorical variables were presented as counts and percentages. We performed comparisons of door-to-clinician times between racial and ethnic groups using ANOVA for normally distributed data and the Kruskal-Wallis test for non-normally distributed data. Chi-square tests were used for categorical variables.

To assess the association between race/ethnicity and door-to-clinician time, we used a generalized linear model with a gamma distribution and log link function to account for the heavily right-skewed outcome. This model estimates adjusted time ratios. These are expressed as rate ratios (RR), representing the proportional change in expected wait time. The model included an intercept term representing the baseline door-to-clinician time when all independent variables were at their reference levels. Variables were selected a priori based on clinical relevance and conceptual considerations. The model included race/ethnicity, sex, ESI level, clinician type, and initial vital signs (systolic blood pressure, heart rate, and respiratory rate). Interaction terms between race/ethnicity and sex, as well as between race/ethnicity and triage level, were tested to explore potential effect modification. We conducted all analyses using SPSS v30 (IBM Corp., Armonk, NY).

## RESULTS

### Patient Selection and Exclusions

We initially identified 4,065 patients. Of those, 139 were excluded: five who had an ESI level of 4; 23 who eloped or left AMA; 42 with missing troponin results; and 69 with missing data on key variables. The final analytic sample included 3,925 patients (Figure).

### Descriptive Stat and Unadjusted Analysis

A total of 3,925 patients were included in the analysis. The majority were White (n = 2,941; 74.9%), followed by Hispanic/Latino (n = 480; 12.2%), Black (n = 213; 5.4%), Native American (n = 135; 3.4%), Asian (n = 96; 2.4%), and unknown (n = 60; 1.5%). There were 1,826 females (46.5%) and 2,099 males (53.5%). Summary statistics stratified by race and ethnicity are presented in Table 1.

The mean age of the overall cohort was 57.9 ± 15.7 years. White patients were significantly older than other racial/ethnic groups (58.9 ± 15.5 years), with Native American patients being the youngest on average (52.5± 13.4 years). Sex distribution varied significantly across racial and ethnic groups (*P* < .01), with Hispanic/Latino patients having the highest proportion of females (53.1%) and Black patients the lowest (36.6%). In terms of acuity, 1,554 patients (39.6%) were triaged as ESI level 2, and 2,371 (60.4%) were triaged as level 3. The distribution of triage levels varied significantly across racial/ethnic groups (*P* = .02). Asian patients had the highest proportion of level 3 triage (72.9%), while White patients had the highest proportion of level 2 (40.7%). Initial vital signs were comparable across all groups with no significant differences.

Regarding the outcome variable (door-to-clinician time/minute), the bivariate analysis showed significant differences across racial/ethnic groups (*P* < .001). The overall median door-to-clinician time for the cohort was 15.9 minutes [interquartile range 8.0–36.0]. Native American patients experienced the longest delays, with a median of 23.8 minutes [13.9–49.8], followed by Asian patients (18.6 minutes [8.4–36.5]) and those with unknown race/ethnicity (18.6 minutes [11.1–36.8]). Hispanic/Latino patients also had longer wait times than the overall median, at 17.1 minutes [9.3–43.7]. In contrast, White and Black patients had shorter median wait times of 14.9 minutes [7.1–33.9] and 15.0 minutes [8.8–38.7], respectively (Table 1).

### Adjusted Analysis Results

In the adjusted analysis using a generalized linear model with a gamma distribution and log link, only the Hispanic/Latino group had a statistically significant association with longer door-to-clinician times. Compared with White patients (adjusted mean 14.9 minutes), Hispanic/Latino patients had an adjusted mean of 18.2 minutes (absolute difference +3.3 minutes; 22% longer wait; adjusted rate ratio 1.22, 95% CI, 1.09–1.36, *P* < .001). Adjusted mean times were also higher for Black (16.5 minutes), Native American (17.7 minutes), and Asian (15.1 minutes) patients relative to White patients, but these differences were not statistically significant. Both rate ratios and adjusted mean times are presented in Table 2.

Among covariates, increasing age was associated with slightly shorter door-to-clinician times (Exp(B) = 0.99, 95% CI, 0.99–0.99, *P* < .001). Triage level had the strongest effect: patients triaged as ESI level 3 waited nearly five times longer than those at level 2 (Exp(B) = 4.90, 95% CI, 1.86–12.94, *P* < .01). Respiratory rate was inversely associated with wait time (Exp(B) = 0.97, 95% CI, 0.96–0.98, *P* < .001), while heart rate showed a small positive association. Both effect sizes were modest, suggesting limited clinical impact. No significant associations were observed for sex, clinician type, or systolic blood pressure (Table 2).

## DISCUSSION

This retrospective observational study examined the association between race/ethnicity and door-to-clinician time in the ED among patients presenting with confirmed acute cardiac chest pain. Using a generalized linear model with a gamma distribution and log link, we found that Hispanic/Latino patients experienced significantly longer wait times compared to White patients, with an estimated 22% (3.3 minutes) increase in door-to-clinician time after adjusting for key demographic and clinical covariates. Although other race/ethnicity groups also had longer average wait times compared to White patients, these differences were not statistically significant. Notably, the effect size for Native American patients approached significance, suggesting a potential disparity warranting further investigation in larger or more diverse cohorts.

While the observed adjusted difference in door-to-clinician time between Hispanic/Latino and White patients was approximately 3.3 minutes (18.2 vs 14.9 minutes), this gap was statistically significant and unlikely to be due to chance (Exp(B) = 1.22, 95% CI, 1.09–1.36, *P* < .001). The clinical relevance of this time difference warrants careful consideration. In isolation, a three-minute delay may appear modest; however, in the context of acute cardiac presentations, even small intervals can influence downstream care. Earlier clinician contact facilitates prompt diagnostic ordering, such as troponin testing, ECG interpretation, and risk stratification, all of which can impact time to intervention and overall outcomes. Moreover, disparities in door-to-clinician time may be part of broader care delays, compounded across sequential steps in ED management, that disproportionately affect historically marginalized groups. Thus, while the absolute time difference may be brief, its significance lies in what it represents: a measurable inequity in initial evaluation that could have cascading effects throughout the patient’s care trajectory.

These findings contribute to the growing body of literature documenting disparities in cardiovascular emergency care by race and ethnicity.^6^,^16^–^19^ Furthermore, our results align with previous research showing that Hispanic/Latino patients experience longer wait times.^10^,^13^ Importantly, the longer wait times observed for Hispanic/Latino patients with confirmed cardiac chest pain likely reflect systemic barriers such as language differences, implicit bias, socioeconomic challenges, structural racism, stereotyping, or limited access to care.^12^,^13^,^16^,^20^–^22^ Although our study did not directly evaluate these underlying factors, the observed disparity underscores the need for targeted interventions to ensure equitable triage and timely clinician evaluation for all patient populations. Importantly, delays in initial evaluation may lead to postponed clinical decision-making, diagnostics, and interventions, particularly in time-sensitive conditions such as ACS, where treatment delays have been associated with increased morbidity and mortality.^23^

Furthermore, additional findings from this study merit attention. Patients triaged at ESI level 3 experienced significantly longer door-to-clinician times than those at level 2, consistent with the intended prioritization of more acute cases. These differences may also reflect local variation in how ESI is applied, as triage decisions can vary between clinicians even within standardized protocols. Initial vital signs were also associated with wait times: higher heart rate was associated with slightly longer wait times, whereas higher respiratory rate was associated with slightly shorter wait times. While these associations were statistically significant, their effect sizes were small, and their clinical relevance should be interpreted with caution.

Conversely, contrary to some prior research reporting longer wait times for Black patients,^7^,^9^,^10^,^21^,^24^ we did not observe significant differences for this group. Differences in local healthcare delivery, patient demographics, or operational factors unique to our setting may explain this discrepancy. Another key factor is our study’s narrower inclusion criteria; we focused specifically on patients with confirmed cardiac chest pain, whereas many prior studies included all patients presenting with chest pain regardless of etiology. This broader inclusion may introduce greater heterogeneity, potentially amplifying observed differences. One strength of our study is the use of a well-defined, clinically homogeneous population, which minimizes misclassification bias and enhances internal validity. Moreover, our use of a generalized linear model with a gamma distribution and log link appropriately modeled the heavily skewed door-to-clinician time, enhancing the accuracy and validity of the results.^25^

Timely evaluation of cardiac chest pain is critical to improving outcomes, and delays during the door-to-clinician interval may lead to missed diagnoses and delayed interventions. Our findings indicate that racial and ethnic disparities in this early phase of care persist even among patients with confirmed cardiac chest pain. This is the first study to explore such disparities in Salt Lake City, Utah, a predominantly White but increasingly diverse metropolitan area.^26^ Our institution’s ED patient population (~25% non-White) is broadly similar, although not identical, to the city’s overall demographics, which should be considered when interpreting generalizability. These results illustrate how structural bias may manifest in less-studied regions and underscore the need for local and national quality improvement efforts to promote equitable ED triage and timely evaluation. Beyond documenting disparities, our findings point to the need for system-level interventions to reduce ED wait times for patients with cardiac chest pain. Practical strategies include the followibg: 1) implementing rapid assessment or clinician-in-triage models to shorten door-to-clinician times; 2) training triage staff in cultural competency and implicit bias mitigation to support equitable prioritization; and 3) leveraging artificial intelligence-based decision support to predict and monitor wait times across demographic groups.

## LIMITATIONS

While these findings offer important insights, several limitations warrant consideration. Residual confounding is possible from unmeasured patient-level factors (eg, language, insurance type, socioeconomic status) and system-level factors. The study overlapped with COVID-19 surges and staffing changes, and we lacked validated crowding indices (National Emergency Department Overcrowding Scale and Emergency Department Working Index), real-time staffing data, arrival mode, and time-of-day/weekend measures. These operational factors may influence wait times and differ by race/ethnicity; thus, findings should be interpreted as associational rather than causal.

Results are based on a single healthcare system, which may limit generalizability, and the study population was relatively homogeneous, with non-White patients comprising ~25%. The small size and under-representation of some racial and ethnic subgroups reduced statistical power, although the absence of disparities in groups such as Black patients may reflect genuinely equitable care at this institution. Finally, we did not capture compliance with triage protocols (eg, timely ECG or standing orders), which may have confounded door-to-clinician time estimates. Multisite studies with larger, more diverse populations and standardized operational measures are needed to confirm these findings and identify effective interventions.

## CONCLUSION

This study demonstrates significant racial and ethnic disparities in door-to-clinician time among patients presenting with confirmed acute cardiac chest pain in the ED. Specifically, Hispanic/Latino patients experienced substantially longer wait times compared to White patients, highlighting ongoing inequities in timely emergency cardiovascular care. Although other groups did not show statistically significant differences, trends among Native American patients suggest potential disparities that merit further investigation. These findings emphasize the critical need for targeted interventions to address systemic barriers and ensure equitable, prompt evaluation for all patients with time-sensitive cardiac conditions. Future research should focus on elucidating underlying causes and developing strategies to reduce these disparities across diverse healthcare settings.

## Figures and Tables

**Figure:**
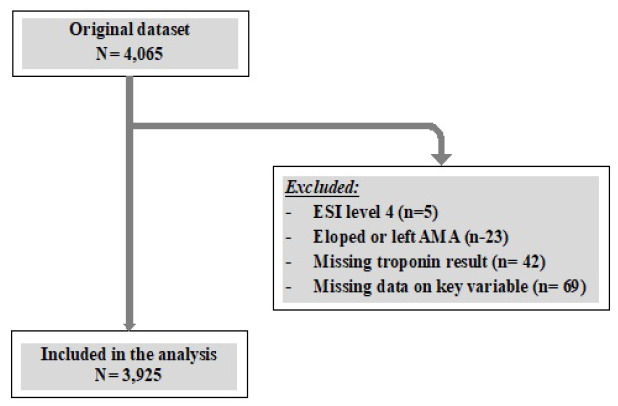
Flow diagram showing inclusion and exclusion criteria for a retrospective study examining door-to-clinician times for cardiac chest pain by race/ethnicity. *AMA*, against medical advice; *ESI*, Emergency Severity Index.

**Table 1:** Baseline demographic and clinical characteristics of patients with confirmed cardiac chest pain, stratified by race/ethnicity (N = 3,925), in a retrospective study of emergency department visits for cardiac chest pain.

Variable	Total (N = 3,925)	White (n = 2,941)	Hispanic/Latino (n = 480)	Black (n = 213)	Native American (n = 135)	Asian (n = 96)	Unknown (n = 60)	P-value
Age (Mean ± SD)	57.9 ± 15.7	58.9 ± 15.5	54.6 ± 16.1	55.8 ± 14.1	52.5 ± 13.4	56.1 ± 16.5	57.9 ± 15.7	< .001
Sex								< .01
Female	1,826 (46.5%)	1,352 (46.0%)	255 (53.1%)	78 (36.6%)	69 (51.1%)	43 (44.8%)	29 (48.3%)	
Male	2,099 (53.5%)	1,589 (54.0%)	225 (46.9%)	135 (63.4%)	66 (48.9%)	53 (55.2%)	31 (51.7%)	
ESI level								.02
Level 2	1,554 (39.6%)	1,198 (40.7%)	173 (36.0%)	90 (42.3%)	45 (33.3%)	26 (27.1%)	22 (36.7%)	
Level 3	2,371 (60.4%)	1,743 (59.3%)	307 (64.0%)	123 (57.7%)	90 (66.7%)	70 (72.9%)	38 (63.3%)	
SBP Mean ± SD	141.8 ± 26.0	141.5 ± 25.9	142.0 ± 24.9	141.5 ± 28.0	145.7 ± 27.9	145.6 ± 30.3	141.5 ± 24.4	.38
HR Mean ± SD	87.4 ± 20.3	87.2 ± 20.2	88.7 ± 20.9	86.2 ± 19.9	90.2 ± 21.5	86.9 ± 20.0	86.7 ± 21.1	.34
RR Mean ± SD	19.0 ± 4.1	19.0 ± 4.0	19.3 ± 4.3	19.5 ± 6.0	19.7 ± 4.6	19.0 ± 3.9	18.5 ± 3.3	.10
Clinician type								.95
ACP	525 (13.4%)	398 (13.5%)	65 (13.5%)	26 (12.2%)	15 (11.1%)	12 (12.5%)	9 (15.0%)	
MD/DO	3,400 (86.6%)	2,543 (86.5%)	415 (86.5%)	187 (87.8%)	120 (88.9%)	84 (87.5%)	51 (85.0%)	
ED LOS/hour Median [IQR]	5.8 [4.3–7.6]	5.8 [4.3–7.6]	5.8 [4.5–8.1]	6.1 [4.7–8.0]	6.6 [4.9–8.4]	5.6 [4.4–7.2]	5.3 [3.9–6.7]	< .001
Door-to-clinician Median [IQR]	15.9 [8.0–36.0]	14.9 [7.1–33.9]	17.1 [9.3–43.7]	15.0 [8.8–38.7]	23.8 [13.9–49.8]	18.6 [8.4–36.5]	18.6 [11.1–36.8]	< .001

*ACP*, advanced care practitioner; *ESI*, Emergency Severity Index; *HR*, heart rate; *IQR*, interquartile range; *LOS*, length of stay; RR, respiratory rate; *MD/DO*, physician; *SBP*, systolic blood pressure.

**Table 2:** Association between race/ethnicity and door-to-clinician time in a retrospective study of adult patients presenting to the emergency department with cardiac chest pain. (Estimates are derived from a generalized linear model (gamma distribution with log link).

Variables	B	*Exp (B)	95% CI	P-value	Adjusted Mean (min)	Difference
Race/Ethnicity						
White (Ref)	—	—	—	—	14.9	—
Hispanic/Latino	0.20	1.22	1.09 – 1.36	< .001	18.2	+3.3
Black	0.10	1.11	0.94 – 1.30	.20	16.5	+1.6
Native American	0.17	1.19	0.97 – 1.43	.09	17.7	+2.8
Asian	0.01	1.01	0.79 – 1.27	.95	15.1	+0.2
Unknown	−0.09	0.91	0.69 – 1.22	.55	13.6	−1.3
Age (Year)	−0.01	0.99	0.99 – 0.99	< .001	—	—
Sex						
Female (Ref)	—	—	—	—	14.9	
Male	−0.03	0.97	0.90 – 1.05	.49	14.5	−0.4
ESI						
ESI level 2 (Ref)	—	—	—	—	14.9	
ESI level 3	1.59	4.90	1.86 – 12.94	< .01	73.0	+58.1
Clinician type						
ACP (Ref)	—	—	—	—	14.9	
MD/DO	−0.01	0.99	0.90 – 1.11	.93	14.8	−0.1
SBP (mm Hg)	0.001	1.00	1.00 – 1.00	.55	—	—
HR (bpm)	0.002	1.00	1.00 – 1.00	.02	—	—
RR (b/min)	−0.03	0.97	0.96 – 0.98	<0.001	—	—

Adjusted means represent model-based expected times (estimated marginal means).

“Difference vs Ref” = adjusted mean – reference group adjusted mean.

Continuous covariates are reported as multiplicative effects per unit; adjusted means are not reported since values depend on selected reference points.

* Exp(B) represents the exponentiated coefficient from the gamma (log-link) model and is interpreted as an adjusted time ratio (rate ratio) for door-to-clinician time. Values >1 indicate a proportional increase (longer expected wait) and values <1 indicate a proportional decrease (shorter expected wait) relative to the reference group; for continuous covariates, the ratio is per 1-unit increase.

*ACP*, advanced care practitioner; *bpm*, beats per minute; *b/min*, breaths per minute; *ESI*, Emergency Severity Index; *HR*, heart rate; *MD/DO*, physician; *mm Hg*, millimeters of mercury; *RR*, respiratory rate; *SBP*, systolic blood pressure.
